# Association of Body Iron Metabolism with Type 2 Diabetes Mellitus in Chinese Women of Childbearing Age: Results from the China Adult Chronic Disease and Nutrition Surveillance (2015)

**DOI:** 10.3390/nu15081935

**Published:** 2023-04-18

**Authors:** Jie Feng, Xiaoyun Shan, Lijuan Wang, Jiaxi Lu, Yang Cao, Lichen Yang

**Affiliations:** 1 Key Laboratory of Trace Element Nutrition of National Health Committee, National Institute for Nutrition and Health, Chinese Center for Disease Control and Prevention, Beijing 100050, China; fengjie@ninh.chinacdc.cn (J.F.); shanxiaoyun0924@163.com (X.S.); wanglj@ninh.chinacdc.cn (L.W.); lujx@ninh.chinacdc.cn (J.L.); yasmine0814@163.com (Y.C.); 2 Hunan Key Laboratory of Typical Environmental Pollution and Health Hazards, School of Public Health, Hengyang Medical School, University of South China, Hengyang 241001, China

**Keywords:** iron storage biomarkers, type 2 diabetes mellitus, association, women of childbearing age

## Abstract

High iron stores have been reported to be associated with type 2 diabetes mellitus (T2DM). However, evidence for the associations of iron metabolism with T2DM is inconsistent, and whether there is a threshold effect remains controversial. In the present study, we aimed to examine the associations between various iron biomarkers and the risk of T2DM as well as impaired glucose metabolism (IGM) and hyperglycemia in Chinese women of childbearing age. A total of 1145 women were divided into three groups (normal blood glucose metabolism group; IGM group; T2DM group). Biomarkers of iron metabolism (serum ferritin (SF), transferrin, soluble transferrin receptor (sTfR), transferrin saturation, serum iron, total body iron, and sTfR-to-lgferritin index) were measured. After adjusting for various confounding risk factors, SF and sTfR were positively associated with the risk of IGM (fourth vs. first quartile: SF odds ratio (OR) = 1.93 (95% CI 1.17–3.20) and sTfR OR = 3.08 (95% CI 1.84–5.14)) and T2DM (SF OR = 2.39 (95% CI 1.40–4.06) and sTfR OR = 3.84 (95% CI 2.53–5.83)). There was a nonlinear relationship between SF and risk of T2DM and hyperglycemia (*p* for nonlinearity < 0.01). Our findings suggested that SF and sTfR could be independent predictors of T2DM risk.

## 1. Introduction

According to the International Diabetes Federation, the global diabetes prevalence in 2019 was 9.3% (463 million people), estimated to rise to 10.2% (578 million) by 2030 and 10.9% (700 million) by 2045 [1]. Type 2 diabetes (T2DM) has become a global pandemic and a threat to human health and global economies. The etiology of T2DM is multi-factorial and not fully demonstrated yet.

Iron is an essential trace element for humans to regulate metabolic processes such as DNA synthesis and oxygen transport. It is involved in the production of reactive oxygen species. Thereby, elevated body iron stores have been reported to cause high levels of oxidative stress and decreased insulin secretory capacity [2]. Iron overload has also been described as a possible cause of T2DM and its comorbidities [3].

Serum ferritin (SF), the most commonly used marker of the body’s iron storage, has been widely reported to be associated with an increased risk of T2DM [4,5]. However, epidemiologic evidence is not consistent, as several studies reported a null association either in the whole population [6] or in women [7]. Furthermore, though some researchers have explored a dose–response relationship between SF and T2DM risk [8,9,10,11], no clear-cut range within which the SF should be maintained was established. 

As SF is affected by many factors, such as inflammation and liver disease and injury [12], other biomarkers of iron metabolism are needed to provide additional information on the role of iron in the pathogenesis of T2DM. Common indicators include transferrin, transferrin saturation (TSAT), soluble transferrin receptor (sTfR), serum iron (SI), etc. For instance, a large prospective European case-cohort study explored the associations of multiple iron biomarkers such as SF, transferrin, TSAT, and SI with incident of T2DM [9]. However, most prior studies were carried out among Western populations [13,14,15], and the results were highly heterogeneous. Moreover, the conclusive evidence for Asian, especially Chinese, populations is limited [16,17]. The role played by iron stores in T2DM in Asian populations has remained poorly understood. Therefore, it is necessary to analyze the relationship between iron stores and T2DM in the Chinese population.

Pregnant women are more sensitive to iron malnutrition and abnormal glucose metabolism, which could lead to an increased risk of adverse pregnancy outcomes. Although previous studies reported that excessive iron was linked to T2DM risk, Li et al. reported that insufficient iron intake could also increase the risk of T2DM in Chinese women [18]. The evidence so far has been inconsistent. Therefore, in the present research, we aimed to assess the associations of multiple biomarkers of iron metabolism, including SF, transferrin, sTfR, SI, TSAT, total body iron (TBI), and sTfR-to-lgferritin (sTfR-F) index, with IGM and T2DM risk in Chinese women of childbearing age, to provide strong evidence to fully clarify the role of iron metabolism in the development of T2DM and better guide iron reference intake in these populations.

## 2. Materials and Methods

### 2.1. Subjects

The present study was based on the data obtained from the database of the China Adult Chronic Disease and Nutrition Surveillance (2015), a nationally representative cross-sectional survey. The study was designed as a case-control study of T2DM patients. A total of 1145 women of childbearing age (18–44 years) with a complete physical examination were selected for the study. Pregnant and lactating women were excluded.

Subjects were classified into three groups (I, II, III) based on their glycemic states according to the WHO criteria [19] (NGM group, FBG < 6.1 mmol/L, N = 381; IGM group, FBG ≥ 6.1 mmol/L but <7.0 mmol/L, N = 353; T2DM group, FBG ≥ 7 mmol/L, or glycated hemoglobin (HbA1c) > 6.5%, N = 411). The sample selection was performed randomly to minimize any bias. 

All of the subjects gave their informed consent for inclusion before they participated in the study. The study was conducted in accordance with the Declaration of Helsinki, and the protocol was approved by the Ethics Committee of the National Institute of Nutrition and Health, Chinese Center for Disease Control and Prevention (file number 201519-B).

### 2.2. Data Collection and Variable Classifications

Physical examinations were performed by trained medical staff following standardized procedures. Height and weight were measured while subjects were wearing light clothing without shoes. BMI was calculated as weight (kg) divided by square of height (m^2^). Waist circumference (WC, cm) was measured by a tape measure with a precision of 0.1 cm. Systolic blood pressure (SBP; mmHg) and diastolic blood pressure (DBP; mmHg) were measured three times, and the average was used in the analysis.

### 2.3. Laboratory Measurements

Fasting venous blood was collected from each participant and divided into an anticoagulation tube and a serum separator tube, separately. The blood samples in the serum separator tube were promptly centrifuged at 3000× *g* for 15 min after blood collection, divided into an aliquot of serum, and frozen at −80 °C for subsequent assays. FBG, high-density lipoprotein cholesterol (HDL-C), low-density lipoprotein cholesterol (LDL-C), total cholesterol (TC), triglyceride (TG), and C-reactive protein (hsCRP) levels were measured by enzymatic methods using an automatic biochemical analyzer (Hitachi 7600, Tokyo, Japan). The SF concentrations were measured by an enzymatic method using the Roche Cobac e601 automatic electrochemiluminescence immunoassay system. Transferrin, sTfR, and α-acid glycoprotein (AAG) concentrations were measured by the Roche Cobac C601 automatic biochemical analyzer (Switzerland). SI concentrations were detected by inductively coupled plasma mass spectrometry (ICP-MS, PerkinElmer, NexION 350, Waltham, MA, USA). In addition, TSAT, TBI, and sTfR-F index were calculated as follows: TSAT = SI (μmol/L)/(transferrin (g/L) × 25.2) [20], TBI = −[log(sTfR/SF)-2.8229]/0.1207 [21], and sTfR-F index = sTfR (mg/L)/lgferritin (ng/mL).

### 2.4. Statistical Analysis

Data management and statistical analyses were performed with IBM SPSS version 23 (SPSS Inc., Chicago, IL, USA) and R version 4.0.3 statistical software, with the smoothHR, survival, rms, Hmisc, and SparseM packages (The Comprehensive R Archive Network: http://cran.rproject.org (accessed on 2 September 2022)). Data are presented as mean with standard deviation (SD) for normally distributed variables, or median with first and third quartiles (P25 and P75) for non-normally distributed variables. Categorical variables are presented as percentages and compared by using the chi-squared test. 

Partial Pearson coefficients adjusted for age, education, physical activity, district, and city type between markers of iron metabolism and risk factors of T2DM were calculated in the overall study sample. 

Multivariate logistic regression was applied to analyze the relationship between iron biomarkers and risk of IGM, T2DM, and hyperglycemia. The levels of SF, sTfR, transferrin, SI, TSAT, TBI, and sTfR-F index were categorized into quartiles. The odds ratios (ORs) and the 95% confidence intervals (95% CIs) were estimated using the lowest quartile as the reference. The crude model was not adjusted. The model was adjusted for age, education, race, smoke, drink, exercise, BMI, WC, SBP, DBP, TC, TG, HDL-C, LDL-C, UA, hemoglobin, and inflammatory markers such as hsCRP and AAG. A p-trend analysis was performed by treating the quartiles as continuous variables in the regression analyses. All of the statistical tests were two-sided, and statistical significance was determined as *p* < 0.05. 

The possible nonlinear relation between iron markers and IGM, T2DM, and hyperglycemia risks was analyzed using restricted cubic spline regression with knots at the 25th, 50th, and 75th percentiles of these markers. 

## 3. Results

### 3.1. Characteristics of the Study Population

Demographic and clinical data of the participants for women of childbearing age are shown in Table 1. Compared with the normal NGM group (32.6 (25.8–40.4) years), subjects of IGM and T2DM groups had higher median ages (39.9 (34.6–43.1) years and 39.4 (34.0–42.8) years, respectively). In addition, subjects in IGM and T2DM groups had higher levels of body mass index (BMI), waist circumference (WC), systolic blood pressure (SBP), diastolic blood pressure (DBP), triglyceride (TG), total cholesterol (TC), low-density lipoprotein cholesterol (LDL-C), and uric acid (UA), but lower levels of high-density lipoprotein cholesterol (HDL-C) compared to those in the NGM group (*p* < 0.001). Inflammatory indicators such as α-acid glycoprotein (AAG) and high-sensitivity C-reactive protein (hsCRP) increased significantly across the spectrum of NGM, IGM, and T2DM. However, no significant differences were observed in the hemoglobin levels between the three different groups (*p* > 0.05). 

### 3.2. Characteristics of the Iron Status

Distribution data of biomarkers of iron metabolism such as SF, transferrin, sTfR, TSAT, SI, TBI, and sTfR-F-index for women of childbearing age are listed in Table 2. SF levels were the highest in the T2DM group, followed by IGM and NGM groups, in order. There were significant statistical differences among the three groups (*p* < 0.01). Similarly, the sTfR levels in IGM and T2DM groups were significantly higher compared with those in the NGM group (*p* < 0.01). On the contrary, TSAT and SI levels in the NGM group were higher than those in IGM and T2DM groups. Among the three groups, TBI levels were the highest in the T2DM group. The concentrations of transferrin in these three groups had no significant difference.

Table 3 summarizes the partial correlation coefficients between biomarkers of iron metabolism and several T2DM risk factors in the overall study sample, adjusted for age, education, physical activity, district, city type, and T2DM status. All investigated iron markers showed strong correlations with each other (*p* < 0.01). Among them, SF levels were positively correlated with SI, TBI, and TSAT, and inversely correlated with sTfR, transferrin, and sTfR-F-index. In addition, components including BMI, WC, and TG were moderately correlated with SF, transferrin, and TBI levels, while none of them correlated with sTfR, SI, and TSAT. Inflammatory biomarkers such as hsCRP and AGG were positively correlated with SF only. Furthermore, FBG was observed to be moderately correlated with SF and sTfR, and HbA1c levels were correlated with transferrin only.

### 3.3. Odds Ratios for IGM, T2DM, and Hyperglycemia in Quartiles of Markers of Iron Storage

To evaluate the associations between levels of iron biomarkers and the risk of IGM, T2DM, and hyperglycemia, multivariate logistic analysis was applied. The results are summarized in Table 4. 

Compared with the lowest SF quartile, the ORs for IGM and T2DM in the highest quartile were 2.09 (95% confidence interval (CI), 1.38–3.17) and 3.60 (2.37–5.48), respectively. The ORs were attenuated but remained significant after adjustment for age, education, race, smoke, drink, exercise, BMI, WC, SBP, DBP, TC, TG, HDL-C, LDL-C, UA, hemoglobin, and inflammatory markers such as hsCRP and AAG (the adjusted OR for IGM and T2DM in the fourth quartile of SF levels were 1.93 (1.17–3.20) and 2.39 (1.40–4.06), respectively).

High levels of sTfR also showed a significant trend towards increased risk of IGM and T2DM. In the adjusted model for sTfR, the ORs for IGM and T2DM in the fourth quartile of sTfR were 3.08 (1.84–5.14) and 2.47 (1.50–4.07), respectively. Finally, in the adjusted model for sTfR-F-index, the ORs for IGM and T2DM were 1.85 (1.12–3.05) and 1.16 (0.70–1.92), respectively.

The same models were employed in evaluating the relationship between the other iron biomarkers and the risk of IGM and T2DM. There were no significant associations between transferrin, SI, TBI, and TSAT levels and the risk of IGM, even after adjusting for confounding factors. However, the fourth quartile of TBI increased the risk of incident of T2DM (OR = 2.36 (1.57–3.53)) and the lower quartiles of SI and TSAT were associated with higher ORs of T2DM (*p* < 0.05). No significant associations between IGM, T2DM, and transferrin were observed in our study. 

**Figure 1:**
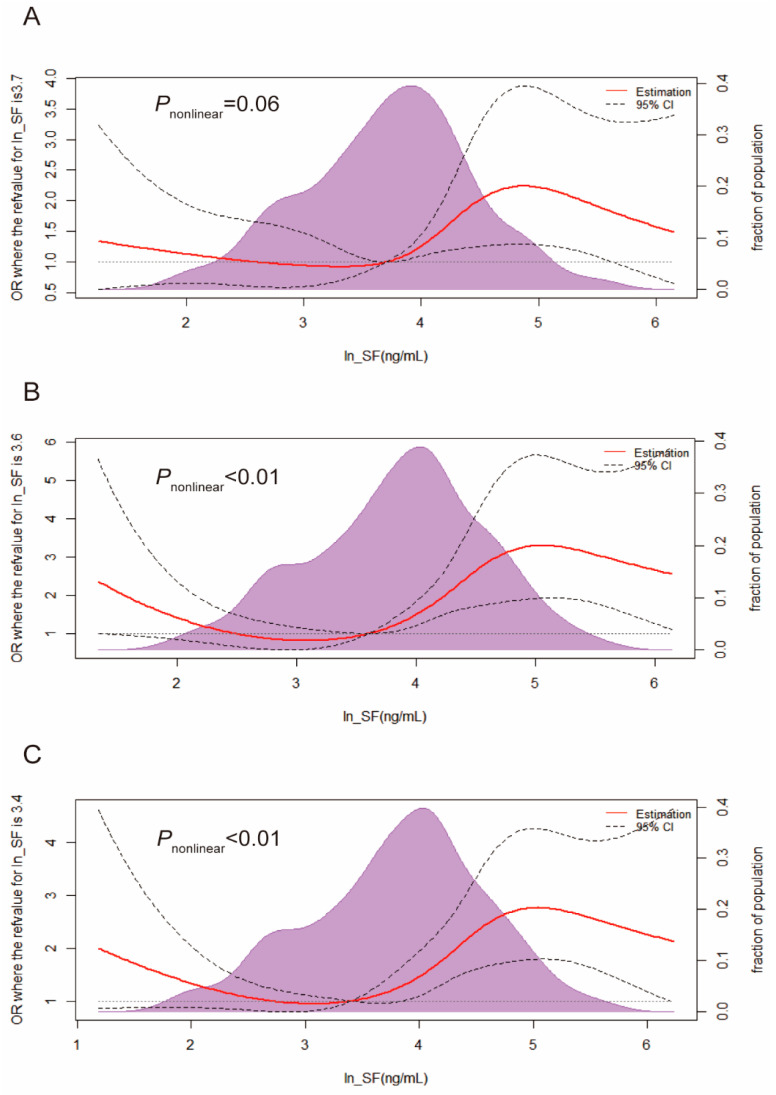
Restricted cubic spline regression analysis for the association between SF and IGM (**A**), T2DM (**B**), and hyperglycemia (**C**). The analysis was adjusted for age, education, race, smoke, drink, exercise, BMI, WC, SBP, DBP, TC, TG, HDL-C, LDL-C, UA, hemoglobin, hsCRP, and AAG. Abbreviation: CI, confidence interval; IGM, impaired glucose metabolism; T2DM, type 2 diabetes mellitus.

In addition, the RCS analysis revealed a nonlinear relationship between SF and risk of T2DM and hyperglycemia (*p* < 0.01 for nonlinearity), but not with IGM risk (*p* = 0.06 for nonlinearity) (Figure 1). This indicated that there was a possible threshold effect for the SF on T2DM and hyperglycemia risk. There was no strong evidence of a nonlinear relationship between SF and risk of IGM.

## 4. Discussion

In the present study, the associations of multiple iron metabolism biomarkers, including SF, transferrin, sTfR, TSAT, SI, TBI, and sTfR-F-index, with IGM, T2DM, and hyperglycemia were investigated. We found that higher SF levels were significantly associated with an increased risk of IGM, T2DM, and hyperglycemia in this research. These associations were slightly attenuated but still significant after adjustment for various confounding risk factors. This finding suggests that SF plays a significant role in T2DM development.

SF regulates iron homeostasis and is a widely used marker for body iron stores [22]. The association between SF and T2DM risk has been extensively studied. With the exception of a few studies that found no association between SF and T2DM risk [6], most cross-sectional and prospective studies have demonstrated a positive association between SF levels and T2DM in different populations [10,23,24,25,26]. It is well known that SF, in addition to reflecting body iron stores, is also influenced by systemic inflammation, which is thought to be involved in the pathophysiological mechanisms underlying diabetes [27]. In the present study, the adjustment of inflammatory markers such as hsCRP and AAG did not materially change the association between SF and IGM, T2DM, and hyperglycemia. Similar results were obtained in a nested case-control study in Japanese individuals [23] and in a French prospective cohort [13] with adjustment for inflammatory markers. These findings showed that the associations between SF with IGM, T2DM, and hyperglycemia were independent of inflammation. Furthermore, metabolic syndrome and obesity were also risk factors for T2DM and cardiovascular diseases [28]. In our study, the positive associations remained significant even after adjustment for components of metabolic syndrome (including SBP, DBP, TG, LDL-C, and HDL-C), BMI, and WC, which excluded confounding factors of metabolic syndrome and obesity. Similar results were also reported in previous studies [10]. Therefore, SF is considered to be an independent risk factor for IGM, T2DM, and hyperglycemia. It should be noted that SF was significantly increased in subjects with IGM compared with NGM subjects, which was consistent with previous reports [29,30]. Therefore, SF could be a useful biomarker in the early monitoring and prevention of T2DM in Chinese women of childbearing age, similar to Western populations [8,13].

Although the relationship between SF and T2DM has been well clarified, the dose–response of SF is still controversial. In a European cohort research, a significant linear dose–response relationship was observed between SF levels and T2DM risk (hazard ratio [95%] in men and women: 1.07 [1.01–1.12] and 1.12 [1.05–1.19] per 100 ng/mL higher SF level, respectively) [9]. Furthermore, the linear dose–response relationship was also observed in a meta-analysis including 10 prospective studies [11]. Different from the above-mentioned results, other studies have reported a nonlinear association only in the highest category. For example, the result of a 4-year cohort study in South Korea showed that T2DM risk increased in healthy men with SF above 200.6 ng/mL at baseline, suggesting a possible threshold effect between SF and T2DM risk [10]. Another cohort study of European men showed a threshold of about 300 ng/mL [31]. In the present study, we observed that T2DM risk increased significantly when the SF levels were higher than 101.4 ng/mL. Given the different results of the studies, the threshold value cannot be determined at present, and more research relying on larger population samples is needed to fully analyze the dose–effect relationship between SF and T2DM.

Given that the essential role of SF in the organism is the storage of iron, the potential mechanism linking elevated SF with T2DM may be described as follows. Iron is a catalyst for reactive oxygen species, such as hydroxyl radicals, leading to oxidative stress. Iron-induced oxidative stress mediates the apoptosis of pancreatic islets with a resulting decrease in insulin secretory capacity [32]. Alternatively, iron-induced oxidative damage in the liver, pancreas, and the muscle may be the main mechanism of insulin resistance [33]. Furthermore, iron accumulation in hepatocytes may cause impaired hepatic insulin extraction and metabolism [34]. Iron may enhance fatty acid oxidation and suppress glucose oxidation in muscle tissue [35] as well as influence insulin function and glucose uptake in adipose tissue [36].

Furthermore, we measured transferrin, sTfR, TSAT, serum iron, TBI, and sTfR-F-index to provide additional information on the role of iron in the pathogenesis of T2DM. sTfR is the truncated form of the transferrin receptor. Since it is closely related to cellular iron uptake [37] and unaffected by the acute-phase response, it has been proposed to be a novel marker of iron status. Low iron stores result in increased sTfR levels [38]. As expected, we observed an inverse correlation between sTfR and SF in our study. However, the sTfR levels in IGM and T2DM groups were significantly higher than those in the NGM group. Elevated sTfR levels were associated with a higher risk of IGM, T2DM, and hyperglycemia, even after adjustment for possible confounding risk factors. Accordingly, TBI, calculated by SF and sTfR, was also found to be positively correlated with T2DM risk. Similar findings have been reported in some, but not all, studies. The Diabetes Prevention Program cohort study observed that high levels of sTfR increased the risk of T2DM among overweight and obese persons with impaired glucose tolerance [14]. In the nested case-control study among Caucasian individuals [39], sTfR was directly associated with the risk of T2DM in obese individuals and inversely associated in non-obese individuals. These studies speculated that sTfR may reflect a chronic pathological state such as obesity that is causally related to the development of T2DM, possibly unrelated to iron stores [14]. Alternatively, experiments in animal models indicated that acute insulin administration could lead to an increase in sTfR concentration [40]. That is, sTfR levels may be affected by mechanisms other than those related to Fe metabolism (such as insulin sensitivity and obesity), and they could be causally linked to T2DM [37]. However, inconsistent research has also been reported. In a cohort study of the Potsdam European Prospective Investigation into Cancer and Nutrition [8] and cohorts of the Cooperative Health Research in the Region of Augsburg [30], sTfR levels were not related to the risk of T2DM. Taken together, the relationship between sTfR and T2DM is complex and not well understood.

Transferrin is the main iron transport protein in blood. If body iron stores are low, circulating transferrin levels increase. Although transferrin is inversely correlated with SF, transferrin is also positively associated with T2DM risk among French and German populations in previous studies [13,30]. The mechanism may be partially explained by the fact that transferrin has an antagonist effect on insulin in vivo [41] and could induce insulin resistance in adipocytes [42,43]. However, in the present study, no significant associations were observed between transferrin and risk of IGM, T2DM, and hyperglycemia. The reason for this discrepancy may be the different ethnic population and sex.

Elevated TSAT (serum iron expressed as a percentage of the total iron binding capacity) is a useful indicator of iron overload. In previous studies, the relationships between TSAT and the risk of T2DM were mostly heterogeneous. Three population-based studies have indicated a significantly higher risk of T2DM if the TSAT is >50% [15]. A prospective European case-cohort study found that elevated TSAT (≥45% vs. <45%) was associated with a lower risk of T2DM in women only [9]. In two other studies, no significant associations were observed between TSAT and T2DM [44,45], in line with our present results. Some of the representative studies mentioned in the discussion above are summarized in Table 5. 

Our study has several strengths. First, we used data from a reliable, nationally representative database. This provided us with a unique opportunity to study the association between the markers of iron metabolism and T2DM in the general population. Second, multiple iron biomarkers were comprehensively assessed to better evaluate the iron status in IGM and T2DM populations and their associations with IGM and T2DM risk. Third, we adjusted for a large variety of possible confounding T2DM risk factors such as blood pressure, BMI, WC, and various metabolic and inflammatory markers. Nevertheless, this study has several limitations. First, due to the inherent limitations of the cross-sectional study design, we cannot imply a causal relationship between the iron metabolism biomarkers with IGM and T2DM. Longitudinal studies are needed to establish the role of iron metabolism in T2DM development. Second, we cannot rule out the confounding effects of adiponectin and liver enzymes such as alanine transaminase (ALT) and gamma-glutamyl transpeptidase (GGT). It has been reported that adiponectin or liver disease may mediate the relationship between iron and glucose metabolism [5,46]. These residual confounding factors may have affected our results. In the future, large prospective studies as well as intervention studies with comprehensive consideration of the influence of relevant factors are warranted to determine whether there is a causal relationship and threshold effect between body iron levels and T2DM.

## 5. Conclusions

In conclusion, we found that SF and sTfR are independently and positively associated with the risk of IGM, T2DM, and hyperglycemia in Chinese women of childbearing age. There is a possible threshold effect for the SF levels on T2DM and hyperglycemia risk. Transferrin and TSAT are not associated with the risk of the IGM, T2DM, and hyperglycemia. These findings have important clinical significance for monitoring iron biomarkers in the development of T2DM and early prevention.

## Figures and Tables

**Table 1:** General characteristics of the study population (*n* = 1145, medians and interquartile ranges (P25–P75)).

Characteristics	Glucose Metabolism Status			*p*-Value
	NGM (*n* = 381)	IGM (*n* = 353)	T2DM (*n* = 411)	
Age (years)	32.6 (25.8–40.4) *	39.4 (34.0–42.8)	39.9 (34.6–43.1)	<0.001
Nationality (%)				0.198
Han	315 (82.7)	305 (86.4)	357 (86.9)	
Ethnic minority	66 (17.3)	48 (13.6)	54 (13.1)	
Education (%)				<0.001
Primary	117 (30.7)	156 (44.2)	187 (45.5)	
Medium	220 (57.7) *	172 (48.7)	176 (42.8)	
Advanced	44 (11.5)	25 (7.1)	48 (11.7)	
Smoking (%)	2 (0.5)	1 (0.3)	4 (1)	0.459
Alcohol drinking (%)	72 (18.9)	72 (20.4)	75 (18.2)	0.746
Physically active (%)	48 (12.6)	43 (12.2)	75 (18.2)	0.026
District				0.025
Eastern	128 (33.6)	98 (27.8)	144 (35)	
Center	124 (32.5)	107 (30.3)	140 (34.1)	
Western	129 (33.9)	148 (41.9) *	127 (30.9)	
City (%)	171 (44.9)	203 (57.5)	243 (59.1)	<0.001
BMI (kg/m^2^)	23.63 (20.96–26.15) *	24.87 (22.49–27.69)	24.99 (22.22–27.94)	<0.001
WC (cm)	78.5 (71.5–85.4) *	81.5 (75.3–88.4)	83.0 (76.4–90.3)	<0.001
Heart rate	77 (71–84) *	80 (74–90)	80 (74–87)	<0.001
Systolic pressure (mmHg)	118 (109–127) *	125 (116–137)	126 (117–138)	<0.001
Diastolic pressure (mmHg)	72 (66–79) *	77 (70–84)	78 (71–86)	<0.001
FBG (mmol/L)	5.07 (4.65–6.01) *	6.34 (6.2–6.57) *	8.04 (7.18–10.35) *	<0.001
HbA1c (%)	4.8 (4.4–5.1) *	4.9 (4.5–5.4) *	6.5 (5.4–7.7) *	<0.001
TC (mmol/L)	4.25 (3.8–4.86) *	4.61 (4.1–5.23)	4.7 (4.12–5.43)	<0.001
TG (mmol/L)	0.86 (0.62–1.23) *	1.26 (0.88–1.96) *	1.54 (0.97–2.29) *	<0.001
HDL-C (mmol/L)	1.31 (1.12–1.52) *	1.2 (1.05–1.42)	1.19 (1–1.41)	<0.001
LDL-C (mmol/L)	2.50 (2.14–3.09) *	2.83 (2.33–3.43)	2.94 (2.44–3.61)	<0.001
UA (μmol/L)	255.4 (212.6–303.25) *	268 (223.45–319.3)	272 (222.3–318.9)	0.018
hsCRP (mg/L)	0.46 (0.19–1.08) *	0.75 (0.31–1.67) *	1.01 (0.36–2.12) *	<0.001
AAG (g/L)	0.51 (0.43–0.62) *	0.58 (0.47–0.67)	0.58 (0.48–0.7)	<0.001
Hb (mmol/L)	142.07 (132.12–151.22)	142.74 (132.12–151.85)	144.66 (132.72–153.67)	0.223

Abbreviations: NGM, normal glucose metabolism; IGM, impaired glucose metabolism; BMI, body mass index; WC, waist circumference; hsCRP, high-sensitivity C-reactive protein; TC, total cholesterol; TG, triglycerides; LDL-C, low-density lipoprotein cholesterol; UA, uric acid; HDL-C, high-density lipoprotein cholesterol; FBG, fasting blood glucose; AAG, α-acid glycoprotein. *, compared with the other two groups. *p* < 0.05.

**Table 2:** Characteristics of the iron status of the studied population (medians and interquartile ranges (P_25_–P_75_)).

Characteristics	Glucose Metabolism Status			*p*-Value
	NGM (*n* = 381)	IGM (*n* = 353)	T2DM (*n* = 411)	
SF (ng/mL)	41.1 (19.5–74.0) *	55.2 (25.2–103.5) *	72.9 (32.7–138.9) *	<0.001
TRF (g/L)	2.60 (2.33–2.87)	2.67 (2.36–2.96)	2.65 (2.33–2.91)	0.182
sTfR (mg/L)	2.20 (1.73–2.74) *	2.63 (2.14–3.32)	2.54 (2.14–3.27)	<0.001
SI (μmol/L)	22.4 (17.7–28.1) a	21.5 (16.6–26.1)	20.9 (16.8–25.6)	0.004
TBI (mg/kg)	34.1 (31.1–36.3)	34.7 (31.3–36.8)	35.5 (32.4–37.7) *	<0.001
TSAT (%)	35.6 (25.9–44.4) a	32.7 (23.7–41.5)	32.7 (24.5–39.5)	0.026
sTfR-F index (units)	1.4 (1.0–1.9) a	1.5 (1.1–2.2)	1.4 (1.1–2.0)	0.002

Abbreviations: NGM, normal glucose metabolism; IGM, impaired glucose metabolism; SF, serum ferritin; TRF, transferrin; sTfR, serum transferrin receptor; SI, serum iron; TSAT, transferrin saturation; TBI, total body iron; sTfR-F index, sTfR-to-log10ferritin index. *, compared with the other two groups; a, comparing NGM group with IGM group. *p* < 0.05.

**Table 3:** Partial Pearson coefficients (adjusted for age, education, physical activity, district, and city type) between markers of iron metabolism and risk factors of T2DM in the overall study sample (*n* = 1145).

	SF ^#^	TRSF ^#^	sTfR ^#^	SI ^#^	TBI ^#^	TSAT ^#^	sTfR-F Index ^#^
SF ^#^	1	−0.49 ‡	−0.31 ‡	0.40 ‡	0.93 ‡	0.54 ‡	−0.72 ‡
TRSF ^#^	−0.49 ‡	1	0.39 ‡	−0.15 ‡	−0.54 ‡	−0.51 ‡	0.52 ‡
STFR ^#^	−0.31 ‡	0.39 ‡	1	−0.32 ‡	−0.63 ‡	−0.43 ‡	0.87 ‡
SI ^#^	0.40 ‡	−0.15 ‡	−0.32 ‡	1	0.46 ‡	0.93 ‡	−0.45 ‡
TBI ^#^	0.93 ‡	−0.54 ‡	−0.63 ‡	0.46 ‡	1	0.61 ‡	−0.92 ‡
TSAT ^#^	0.54 ‡	−0.51 ‡	−0.43 ‡	0.93 ‡	0.61 ‡	1	−0.58 ‡
sTfR-F index ^#^	−0.72 ‡	0.52 ‡	0.87 ‡	−0.45 ‡	−0.92 ‡	−0.59 ‡	1
AAG ^#^	0.12 ‡	0.19 ‡	0.13 ‡	0.003	0.05	−0.07 †	0.03
hsCRP ^#^	0.22 ‡	0.01	0.05	−0.008	0.16 ‡	−0.01	−0.07 †
FBG ^#^	0.13 ‡	0.02	0.13 ‡	0.03	0.06	0.02	0.02
HbA1c ^#^	0.05	0.08 ‡	0.02	0.04	0.03	0.005	−0.01
UA ^#^	0.26 ‡	−0.09 ‡	−0.01	0.08 †	0.22 ‡	0.10 ‡	−0.14 ‡
BMI ^#^	0.10 ‡	0.16 ‡	0.008	0.05	0.08 ‡	−0.02	−0.04
WC ^#^	0.12 ‡	0.17 ‡	0.05	0.07 †	0.09 ‡	−0.001	−0.03
TC/HDL ^#^	0.24 ‡	0.02	−0.02	0.05	0.20 ‡	0.04	−0.13 ‡
TG ^#^	0.18 ‡	0.08 †	0.06	0.05	0.13 ‡	0.02	−0.05
SBP ^#^	0.06 †	0.11 ‡	0.09 ‡	0.04	0.02	−0.01	0.03
DBP ^#^	0.03	0.17 ‡	0.07 †	0.06	0.003	−0.02	0.03

^#^ variables log-transformed; † *p* < 0.05; ‡ *p* < 0.01.

**Table 4:** Association between markers of iron metabolism and IGM, T2DM, or hyperglycemia.

Iron Markers	ORs (95% CI) for IGM (*n* = 353)			ORs (95% CI) for T2DM (*n* = 411)			ORs (95% CI) for Hyperglycemia (*n* = 764)		
	Quartile 2	Quartile 3	Quartile 4	Quartile 2	Quartile 3	Quartile 4	Quartile 2	Quartile 3	Quartile 4
SF (ng/mL)	20.9–46.4	46.4–87.1	>87.1	22.9–53.9	53.9–101.35	>101.35	23.7–54.7	54.7–102.5	>102.5
Crude Model	1.05 (0.69–1.58)	1.32 (0.87–1.99)	2.09 (1.38–3.17)	0.88 (0.59–1.32)	1.66 (1.12–2.47)	3.60 (2.37–5.48)	1.00 (0.72–1.40)	1.56 (1.11–2.20)	3.13 (2.14–4.58)
Model 1	0.97 (0.61–1.60)	1.29 (0.79–2.11)	1.93 (1.17–3.20)	0.76 (0.47–1.24)	1.62 (0.99–2.64)	2.39 (1.40–4.06)	0.91 (0.61–1.34)	1.54 (1.02–2.32)	2.29 (1.45–3.61)
transferrin (g/L)	2.34–2.63	2.63–2.92	>2.92	2.33–2.63	2.63–2.89	>2.89	2.34–2.64	2.64–2.91	>2.91
Crude Model	0.911 (0.60–1.38)	1.12 (0.74–1.68)	1.39 (0.92–2.09)	0.70 (0.47–1.04)	1.13 (0.76–1.68)	1.09 (0.73–1.61)	0.79 (0.56–1.12)	1.16 (0.81–1.65)	1.21 (0.86–1.72)
Model 1	0.69 (0.43–1.10)	0.67 (0.42–1.08)	0.84 (0.52–1.36)	0.53 (0.33–0.85)	0.84 (0.52–1.36)	0.66 (0.40–1.08)	0.66 (0.44–0.98)	0.87 (0.58–1.31)	0.76 (0.50–1.16)
sTfR (mg/L)	1.93–2.38	2.38–3.05	>3.05	1.93–2.39	2.39–3.04	>3.04	1.98–2.44	2.44–3.11	>3.11
Crude Model	2.37 (1.53–3.67)	3.62 (2.34–5.61)	4.48 (2.87–6.98)	2.08 (1.38–3.13)	3.60 (2.38–5.46)	3.84 (2.53–5.83)	2.09 (1.49–2.92)	3.32 (2.33–4.74)	3.89 (2.70–5.60)
Model 1	1.80 (1.10–2.95)	2.75 (1.68–4.48)	3.08 (1.84–5.14)	1.51 (0.93–2.45)	2.35 (1.44–3.84)	2.47 (1.50–4.07)	1.65 (1.12–2.43)	2.42 (1.61–3.62)	2.76 (1.80–4.25)
SI (μmol/L)	17.2–21.9	21.9–27.3	>27.3	17.1–21.6	21.6–26.8	>26.8	17.0–21.5	21.5–26.7	>26.7
Crude Model	0.81 (0.54–1.22)	0.96 (0.64–1.44)	0.67 (0.44–1.01)	0.94 (0.63–1.40)	0.96 (0.65–1.43)	0.57 (0.38–0.84)	0.91 (0.64–1.30)	1.02 (0.71–1.45)	0.61 (0.43–0.87)
Model 1	0.82 (0.51–1.32)	1.13 (0.70–1.83)	0.64 (0.39–1.05)	0.97 (0.60–1.56)	0.89 (0.55–1.44)	0.58 (0.35–0.95)	0.86 (0.57–1.30)	0.98 (0.64–1.48)	0.58 (0.38–0.88)
TBI (mg/kg)	31.2–34.3	34.3–36.6	>36.6	31.5–34.9	34.9–37.2	>37.2	31.5–34.8	34.8–37.1	>37.1
Crude Model	0.95 (0.63–1.43)	1.11 (0.74–1.66)	1.31 (0.86–1.98)	0.97 (0.65–1.44)	1.47 (0.99–2.18)	2.36 (1.57–3.53)	0.91 (0.65–1.28)	1.18 (0.84–1.67)	1.84 (1.28–2.64)
Model 1	0.81 (0.50–1.31)	1.08 (0.66–1.75)	1.32 (0.80–2.19)	0.92 (0.57–1.49)	1.50 (0.91–2.48)	1.87 (1.12–3.14)	0.84 (0.56–1.25)	1.11 (0.73–1.69)	1.53 (0.98–2.37)
TSAT (%)	25.0–34.3	34.3–42.9	>42.9	25.3–33.7	33.7–42.1	>42.1	24.8–33.5	33.5–41.8	>41.8
Crude Model	0.85 (0.56–1.28)	0.78 (0.52–1.17)	0.63(0.42–0.95)	0.98 (0.66–1.46)	0.88 (0.59–1.30)	0.57 (0.39–0.85)	0.93 (0.65–1.33)	0.85 (0.60–1.22)	0.61 (0.43–0.86)
Model 1	0.93 (0.57–1.49)	1.10 (0.68–1.78)	0.77 (0.47–1.25)	1.21 (0.75–1.96)	1.02 (0.63–1.66)	0.74 (0.45–1.20)	0.97 (0.64–1.47)	1.04 (0.68–1.57)	0.72 (0.48–1.09)
sTfR-F index	1.08–1.44	1.44–2.05	>2.05	1.06–1.38	1.38–1.91	>1.91	1.08–1.42	1.42–2.01	>2.01
Crude Model	1.60 (1.05–2.42)	1.45 (0.96–2.19)	1.96 (1.30–2.97)	1.51 (1.02–2.24)	1.10 (0.74–1.63)	1.33 (0.90–1.98)	1.52 (1.07–2.15)	1.19 (0.84–1.67)	1.60 (1.13–2.26)
Model 1	1.31 (0.82–2.09)	1.07 (0.66–1.73)	1.85 (1.12–3.05)	1.15 (0.72–1.83)	0.73 (0.45–1.17)	1.16 (0.70–1.92)	1.23 (0.83–1.83)	0.88 (0.59–1.31)	1.51 (0.99–2.32)

All results came from multivariable logistic regression analysis. Model 1: adjusted for age, education, race, smoke, drink, exercise, BMI, WC, SBP, DBP, TC, TG, HDL-C, LDL-C, UA, hemoglobin, hsCRP, and AAG. The ORs and the 95% CIs were estimated using quartile 1 as the reference.

**Table 5:** Characteristics of study on the association between iron metabolism and T2DM risk.

Study (First Author, Year)	Study Design	Country/ Ethnicity	Age (Years)	N (T2DM/ Control)	Sex (% Female)	T2DM Diagnosis and Criteria	Iron Biomarkers	Main Findings
Forouhi 2007 [31]	Case-control	U.K.	40–74	360/758	42%	Self-report or HbA1c level > 7%	Ferritin	Ferritin was an independent predictor of the development of T2DM.
Rajpathak 2009 [14]	Nested case-control	USA	≥25	280/280	63.6%	Standardized criteria of the American Diabetes Association and the World Health Organization	Ferritin, sTfR	Elevated sTfR levels were associated with increased T2DM risk among overweight and obese individuals with impaired glucose tolerance. Ferritin levels were not statistically different.
Jung 2013 [10]	Cohort	Korean	23–82	186/1843	Only men	Self-report or FPG ≥ 126 mg/dl or HbA1c ≥ 6.5%	Ferritin	Elevated level of ferritin at baseline was associated with incident T2DM in an Asian population.
Yeap 2014 [5]	Cross-sectional	Australia	17–97	263/3922	56%	Self-report, FPG ≥ 7 mM, or taking glucose-lowering medications	Ferritin, iron, transferrin saturation	Higher ferritin levels were independently associated with prevalent T2DM. Neither iron nor transferrin saturation were associated with T2DM risk in men or women.
Huth 2015 [30]	Cross-sectional	German	25–74	315/2099	51.7%	1999 WHO criteria	Ferritin, transferrin, sTfR, TSAT, sTfR-F index, iron	Ferritin and transferrin were positively associated with IGM and T2DM. TSAT and iron were inversely associated with T2DM.
Podmore 2016 [9]	Cohort	European	>40	11052/14061	Not given	Self-report, linkage to primary care registers, or hospital admissions	Ferritin, TSAT, serum iron, transferrin	Higher ferritin and transferrin were associated with higher risk of T2DM in men and women. Elevated TSAT was associated with lower risk of T2DM in women, but not in men. Serum iron was not associated with T2DM.
Wang 2017 [24]	Case-control	Singapore, Chinese men and women	45–74	485/485	56.3%	Self-report questionnaire	Ferritin	Elevation of ferritin was significantly associated with increased risk of T2DM.
Akter 2017 [23]	Cohort	Japan	51	327/641	10%	American Diabetes Association criteria for the diagnosis of diabetes	Ferritin	Elevated serum ferritin levels were associated with a significantly increased risk of T2DM.
Gao 2022 [26]	Cohort	China	>19	75/5704	54.18%	Self-reported questionnaire	Ferritin	Iron overload increased the risk of T2DM and the association is sex-specific.
Current study	Case-control	China	18–44	411(T2DM)/353 (IGM)/381 (control)	Only women of childbearing	1999 WHO criteria	Ferritin, transferrin, sTfR, SI, TSAT, TBI, sTfR-to-lgferritin index	Ferritin and sTfR were independently positively associated with the risk of IGM, T2DM and hyperglycemia.

## Data Availability

Not applicable.
